# Brief internet-delivered cognitive-behavioural intervention for children and adolescents with symptoms of anxiety and depression during the COVID-19 pandemic: a randomised controlled trial protocol

**DOI:** 10.1186/s13063-022-06836-2

**Published:** 2022-10-22

**Authors:** Caio Borba Casella, Pedro Fonseca Zuccolo, Luisa Sugaya, Aline Santana de Souza, Luara Otoch, Fernanda Alarcão, Wagner Gurgel, Daniel Fatori, Guilherme V. Polanczyk

**Affiliations:** Department and Institute of Psychiatry, Hospital das Clínicas, University of São Paulo School of Medicine, São Paulo, Brazil

**Keywords:** Children and adolescents, Anxiety, Depression, Stress, Irritability, Cognitive-behavioural therapy, Telemedicine, Irritability, COVID-19 pandemic

## Abstract

**Background:**

The COVID-19 pandemic has had major impacts in many different spheres, including mental health. Children and adolescents are especially vulnerable because their central nervous system is still in development and they have fewer coping resources than do adults. Increases in the prevalence of depressive and anxiety symptomatology have been reported worldwide. However, access to mental health care is limited, especially for the paediatric population and in low- and middle-income countries. Therefore, we developed a brief internet-delivered cognitive-behavioural intervention for children and adolescents with symptoms of anxiety and depression. The aim of this proposed study is to test the efficacy of the intervention.

**Methods:**

We will conduct a two-arm, parallel randomised controlled trial involving children and adolescents (8–11 and 12–17 years of age, respectively) with symptoms of anxiety, depression or both, according to the 25-item Revised Child Anxiety and Depression Scale (*t*-score > 70). A total of 280 participants will be randomised to the intervention group or the active control group, in a 1:1 ratio. Those in the intervention group will receive five weekly sessions of cognitive-behavioural therapy via teleconference. The sessions will focus on stress responses, family communication, diaphragmatic breathing, emotions, anger management, behavioural activation and cognitive restructuring. Participants in both groups will have access to 15 videos covering the same topics. Participant-guardian pairs will be expected to attend the sessions (intervention group), watch the videos (control group) or both (intervention group only). A blinded assessor will collect data on symptoms of anxiety, depression and irritability, at baseline, at the end of the intervention and 30 days thereafter. Adolescents with access to a smartphone will also be invited to participate in an ecological momentary assessment of emotional problems in the week before and the week after the intervention, as well as in passive data collection from existing smartphone sensors throughout the study.

**Discussion:**

Internet-delivered interventions play a major role in increasing access to mental health care. A brief, manualised, internet-delivered intervention might help children and adolescents with anxiety or depressive symptomatology, even outside the context of the COVID-19 pandemic.

**Trial registration:**

ClinicalTrials.gov NCT05139433. Registered prospectively in November 2021. Minor amendments made in July 2022.

## Administrative information

Note: the numbers in curly brackets in this protocol refer to SPIRIT checklist item numbers. The order of the items has been modified in order to group similar items (see http://www.equator-network.org/reporting-guidelines/spirit-2013-statement-defining-standard-protocol-items-for-clinical-trials/).

**Table:** 

Title {1}	Brief internet-delivered cognitive-behavioural intervention for children and adolescents with symptoms of anxiety and depression during the COVID-19 pandemic: a randomised controlled trial protocol
Trial registration {2a and 2b}	ClinicalTrials.gov ID: NCT05139433 -Brief Internet-delivered Intervention for Children and Adolescents With Anxiety and Depression Symptoms
Protocol version {3}	Version 1.0 registered in November 2021 Version 1.1 registered in July 2022
Funding {4}	This project is funded by the Fundação de Amparo à Pesquisa do Estado de São Paulo (FAPESP)
Author details {5a}	1—Departamento de Psiquiatria, Faculdade de Medicina FMUSP, Universidade de São Paulo, São Paulo, SP, BR
Name and contact information for the trial sponsor {5b}	Fundação de Amparo à Pesquisa do Estado de São Paulo (FAPESP) converse2@fapesp.br + 55 (11) 3838–4000
Role of sponsor {5c}	None of the project partners involved in the trial have any potential conflicts of interest to declare. None of the funding sources were involved in the study design, the collection of data or the decision to submit this article for publication, nor will be involved in the analysis/interpretation of the data, the writing of the final report or the decision to submit it for publication.

## Introduction

### Background and rationale {6a}

The coronavirus disease 2019 (COVID-19) pandemic has had major impacts on many different aspects of health, including mental health [1]. Because their brains are still in development, children could present short- and long-term physiological, cognitive and behavioural changes related to overly challenging situations such as those produced by the pandemic [2]. Their routine suffered major changes due to the restrictions imposed on school and leisure activities, leading to an increase in sedentary behaviours and screen exposure, as well as a decrease in the number of opportunities to socialise with their peers [3]. That could result in loneliness, which is associated with worse mental health outcomes and damages that can persist for years [4]. Financial losses and unemployment can increase the level of stress, as well as the frequency of anxiety, depression, substance abuse and violent behaviour, among parents, which can have an indirect effect on the mental health of the children and adolescents in the home [5, 6].

The emotional and behavioural effects that the pandemic has had on children and adolescents have been well documented [7–12]. Increases in the prevalence of depressive and anxiety symptomatology have been reported in various countries, including China [13, 14], Germany [10], India [8] and the USA [15], as have increases in hyperactivity, social conflict, conduct disturbances and other behavioural changes [7, 10].

Brazil is one of the countries most severely affected by the pandemic. In May 2021, over one in four COVID-19-related deaths occurred in Brazil [16]. It is also one of the countries in which schools were fully closed for an extended period of time [17]. It is estimated that approximately 4 million students dropped out of school during the pandemic in Brazil [18]. In a previous study conducted by our group [19], we found that, among children and adolescents in Brazil, one in three presented with clinical symptoms of anxiety or depression during the pandemic, and that number remained relatively stable from June 2020 to June 2021. In this context, it is vital to develop initiatives to mitigate the potential impact on this population. Some interventions have been shown to reduce the severity of the psychopathology related to traumatic events, such interventions including trauma-focused cognitive-behavioural therapy (CBT), prolonged exposure for post-traumatic stress disorder and cognitive therapy for post-traumatic stress disorder [20]. In the paediatric population, CBT is a well-established therapy for anxiety and depression [21]. In a meta-analysis, Kramer and Landolt [22] suggested that trauma-focused interventions for children and adolescents should include psychoeducation, teaching of coping skills, parental involvement and some form of trauma exposure. The current scenario, however, presents challenges and obstacles to the implementation of such therapeutic strategies. Brazil is a country of continental proportions, with many remote areas and a limited number of mental health professionals, most of whom reside in the major metropolitan areas [23]. A huge number of children and adolescents are now in need of mental health care, which was already difficult to access before the pandemic [24, 25]. During the pandemic, fear of contamination aggravated that scenario, as did the social distancing mandates and the temporary closure of some supporting services. Fortunately, online access to mental health services has now become more common [26]. Even though most studies of telemental health care have involved adult patients, it has been shown to be applicable in the paediatric population as well [27]. Therefore, telepsychotherapy could be a valuable tool during the COVID-19 pandemic and in other contexts in the future.

We developed a brief cognitive-behavioural intervention, to be delivered online, that is focused on anxiety and depressive symptomatology in children and adolescents. We aimed to craft a structured, user-friendly manual that will be freely available and can be followed by mental health professionals with limited experience and will therefore be disseminated throughout Brazil. Here, we describe the protocol for a randomised controlled trial designed to test the efficacy of this intervention.

### Objectives {7}

The objective of the proposed study is to test the efficacy of a brief cognitive-behavioural intervention to be implemented via teleconference (or telephone call) with a child or adolescent, together with a guardian. In a randomised controlled trial, the efficacy of the intervention will be compared with that of a video-based psychoeducational intervention (active control). We expect the active intervention to be the more effective in reducing emotional symptoms and in improving global functionality.

### Trial design {8}

We will conduct a single-blind, two-arm, parallel-group, superiority randomised controlled trial. Participants will be randomly allocated to the intervention group or to the active control group, in a 1:1 ratio.

## Methods: participants, interventions and outcomes

### Study setting {9}

Participants from all over Brazil will be able to participate remotely. The research centre is the Institute of Psychiatry of the Hospital das Clínicas, operated by the University of São Paulo School of Medicine, in the city of São Paulo, Brazil. The intervention will preferably be administered by videoconference. However, when that is not possible (due to internet connectivity issues or other problems), the sessions will be delivered by telephone.

### Eligibility criteria {10}

Children and adolescents with the following characteristics will be eligible to participate in the study: being between 8 and 17 years of age; living in Brazil; having a total *t*-score of ≥ 70 on the 25-item version of the Revised Children’s Anxiety and Depression Scale, parent report (RCADS-25-P) [28]; and having a total *t*-score ≥ 70 on the RCADS-25, child report (RCADS-25-C) [29]. The RCADS-25 measures the frequency of symptoms of anxiety and depression, using a 4-point Likert scale (“never”, “sometimes”, “often” or “always”), with subscale scores (for anxiety and depression, respectively) and an overall score. In an international consensus, the RCADS-25 was recommended as an outcome measure for anxiety and depression symptomatology in children and adolescents [30].

Participants will be excluded if there has been no contact between the child/adolescent and the guardian in the 15 days preceding the intervention; if there is not at least one parent/guardian participating in each session (intervention group) or available to watch the videos along with the child/adolescent (active control group); if the guardian is unable to understand the scales used in the assessments or the content of the interventions, according to the judgement of a clinical psychologist; or if the child/adolescent has previously been diagnosed with an autism spectrum disorder, schizophrenia or intellectual disability or has clinical signs of a severe mental disorder or social condition that requires more intensive assessment and treatment, such as severe mood disorder, suicide risk, intense intra-familiar conflict or intense maltreatment victimisation, according to the judgement of a clinical psychologist. Suicidality will be assessed with the aid of the parent-report four-item composite version of the Mood and Feelings Questionnaire for Suicidal Ideation (MFQ-SI) [31, 32], which comprises the following items (marked as “not true”, “sometimes true” or “true”, in relation to the last two weeks): “S/he thought that life wasn’t worth living”; “S/he thought about death or dying”; “S/he thought his/her family would be better off without him/her”; and “S/he thought about killing him/herself”. Children and adolescents whose parents marked the final statement as “true” or “sometimes true” will be excluded from the study and referred for appropriate care that is more intensive. Individuals who have been treated with any psychiatric medication or psychotherapy in the last 30 days will also be excluded.

### Who will take informed consent? {26a}

Parents who are interested in participating in the study will complete an online screening form. If their child is considered eligible in that initial assessment, a research manager will contact them by telephone to explain the study in detail and answer any questions they might have. The informed consent form (for the parent) and the informed assent form (for the child or adolescent) will then be sent, and a videoconference with an assessor will be scheduled, to confirm the eligibility criteria and perform the baseline (T0) evaluation. The forms will be collected at the beginning of this videoconference.

### Additional consent provisions for collection and use of participant data and biological specimens {26b}

Participants will be asked if they agree to allow their data to be used should they choose to withdraw from the trial. We will also ask their permission for the research team to share relevant data with other people at the University of São Paulo or at regulatory agencies, where relevant. This trial will not involve the collection of biological specimens.

## Interventions

### Explanation for the choice of comparators {6b}

Psychoeducational interventions consist of strategies to provide information and tools on how to better live and cope with a condition [33]. There is a growing body of literature on these kinds of interventions in mental health [33, 34], even within the paediatric population [34–36]. There is evidence that such interventions have beneficial effects, resulting in favourable mental health and well-being outcomes in adolescents with depressive symptoms [36]. The choice of a psychoeducational intervention as the comparator is therefore justified.

### Intervention description {11a}

#### Intervention group

We have developed a brief standardised, manualised cognitive-behavioural intervention for treating anxiety and depression in children and adolescents, to be implemented by trained psychologists via teleconference or telephone call. The main focus of the sessions will be skills training, with a CBT approach that employs vocabulary adapted to the target population. The psychotherapy programme will consist of five structured weekly sessions. The structure of each session is described in detail in the manual. Below is a brief description of the topics covered in each psychotherapy session.

- Session 1—The initial session covers the following: identification of the main symptoms and problems; psychoeducation on stress responses; strategies for improving communication within the family (e.g. defining minimal routines at home and how parents can listen to their child empathically); and relaxation and mindfulness techniques (diaphragmatic breathing exercises).
- Session 2—The second session focuses on emotion recognition and anger management. There is a psychoeducational component on emotions, covering the basic emotions of happiness, sadness, fear, disgust, surprise and anger, as well as their usefulness (e.g. motivating us to take necessary actions) and pitfalls (e.g. the potential for suffering). There is a greater emphasis on anger, exploring its physical, cognitive and behavioural components, and on strategies for coping with outbursts of anger, such as how they can be prevented and how to recover from one.
- Session 3—The third session focuses on behavioural activation, which is a technique based on the idea that avoidance behaviours exacerbate depressive symptoms. The technique is aimed at promoting adaptive behaviours that would be positively reinforced by scheduling potentially enjoyable and meaningful activities [37]. The rationale of behavioural activation is to stimulate participation. The therapist will guide the child/adolescent, with the help of a guardian, in creating a brief list of activities to be performed in the following week.
- Session 4—The aim of the fourth session is to teach the principles of cognitive restructuring. To that end, there is a psychoeducational component on what “thoughts” are, how to recognise them and the effects they might have on us, covering the basics of the cognitive model developed by Aaron T. Beck [38]. The last part of the session will focus on Socratic questioning [39].
- Session 5—The final session is aimed at revising any content of the previous sessions that was not clear to the family and providing a referral if necessary (e.g. to the public health care system if the child/adolescent is still symptomatic).

The first four sessions all have a homework component, encouraging the family to practice the strategies between sessions. Each session starts by reviewing the previous week’s homework. As the focus of the intervention is on skills building, the child/adolescent and the main guardian (preferably a parent) will both be required to participate in all sessions. It is expected that parent/guardian participation will improve learning because the adult should act as a model for the child/adolescent on how (and when) to employ the techniques learned. Some of the interventions are also focused on teaching the guardians new skills (such as establishing better routines at home and listening empathically to their child).

The intervention also comprises 15 psychoeducational videos that revise and deepen the content of the sessions. Families are expected (and encouraged) to watch the videos between sessions. The topics of each video are as follows:

• Video 1—general information regarding the project and psychoeducation on stress responses
• Video 2—parenting, communication and cooperation between family members
• Video 3—diaphragmatic breathing
• Video 4—a version of the diaphragmatic breathing video adapted for children
• Video 5—progressive muscle relaxation
• Video 6—mindfulness
• Video 7—psychoeducation on emotions
• Video 8—fear, sadness and anxiety
• Video 9—coping with irritability
• Video 10—behavioural activation
• Video 11—establishing routines
• Video 12—use of electronics by children and adolescents
• Video 13—sleep hygiene
• Video 14—cognitive restructuring
• Video 15—coping with an anxiety attack

Each video is approximately 5 min in length. The families will have access to them via the COMVC smartphone application [40], which is available for Android and iOS. Videos will be made available according to the following schedule: first week, videos 1 to 6; second week, videos 7 to 9; third week, videos 10 to 13; and fourth week, videos 14 and 15.

#### Active control group

Children and adolescents in the active control group will receive a psychoeducational intervention that consists of the same 15 videos described above. The videos will be made available through the same smartphone application and on to the same schedule described for the intervention group.

### Criteria for discontinuing or modifying allocated interventions {11b}

Patients requiring a more intense type of treatment, such as individual psychotherapy or psychotropic medication, because of an increase in symptomatology (e.g. emergence of suicidal ideation), will be referred to the appropriate facility.

If a participant in the intervention group misses a session, the team will attempt to reschedule it, respecting the following criteria: no more than 4 weeks should elapse between the first and last session, and there should be at least 48 h between sessions, in order to allow families enough time to carry out the proposed homework. The fifth session can also be used to cover any topic that was missed because of participant inability to attend a previous session.

### Strategies to improve adherence to interventions {11c}

The intervention sessions will be recorded on video; 10% will be randomly selected to be watched by one of the authors of the manual, to assess therapist adherence to the protocol. Each session is described in detail in the manual, helping make the intervention more homogeneous among the various therapists of the research group. All of the therapists will receive weekly supervisory sessions led by senior psychotherapists. An assessor blinded to the allocation will register the percentage of psychoeducational videos watched by the patient and guardian.

### Relevant concomitant care permitted or prohibited during the trial {11d}

Participants who have, in the last 30 days, undergone any other form of psychotherapy or used any psychotropic medications will be excluded. However, those who present worsening of their symptoms during the intervention and require more intensive support (such as another form of psychotherapy and/or psychotropic medications) will be allowed to complete the protocol.

### Provisions for post-trial care {30}

Participants judged by the psychotherapist or the blinded assessor to be in need of further treatment after the end of the protocol will be referred to an appropriate facility within the Brazilian public health care system.

### Outcomes {12}

The primary outcome measure will be a change in the symptoms of anxiety and depression, as determined by the RCADS-25-P and RCADS-25-C [28, 29], between study entry (T0) and 1–3 days after the end of the intervention (T1, after five sessions of psychotherapy, five weeks of access to the psychoeducational videos, or both). The RCADS-25-P and RCADS-25-C will be completed by an independent clinician blinded to the allocation, on the basis of interviews (through videoconference or telephone call) with the guardians and the children/adolescents, respectively. The response variables will be the continuous T-scores for the three RCADS-25 domains (total, depression and anxiety).

Secondary outcomes will include the following: a change in the symptoms of anxiety and depression at follow-up; global functioning, assessed by the Clinical Global Impression (CGI) scale [41] and the Children’s Global Assessment Scale (CGAS) [30, 42]; the impact of mental health symptomatology, as determined with the Strengths and Difficulties Questionnaire (SDQ) impact supplement [43]; changes in irritability; emotional problems; environmental changes, as determined by passive smartphone data collection; the level of parental satisfaction with the telepsychotherapy sessions; and the potential harm (emergence of suicidal ideation and worsening of the quality of family relationships).

The blinded assessor will evaluate the difference between the anxiety and depressive symptomatology observed at T0 and that observed at follow-up (T2, 30 days after the last psychotherapy session, for the intervention group, or 30 days after the last video is made available, for the active control group) in an interview with the guardian and the child/adolescent, using the RCADS-25-P and RCADS-25-C.

The blinded assessor will evaluate the global functioning of the participants in interviews with the guardian and the child/adolescent (through videoconference or telephone call). The CGI-Severity (CGI-S) and CGAS scores obtained at T0 will be compared with those obtained at T1 and with those obtained at T2. Scores on the CGI-S range from 1 to 7, higher scores corresponding to greater symptom intensity. Scores on the CGAS range from 0 to 100, higher scores indicating better functionality. The CGI-Improvement (CGI-I) scores will be assessed at T1 and T2. Scores on the CGI-I range from 1 (“very much improved”) to 7 (“very much worse”).

The impact of the mental health symptomatology on the lives of the children/adolescents will be compared between T0 and T1, as well as between T0 and T2, by using the SDQ impact supplement [43], the score on which ranges from 0 to 10, higher scores indicating greater distress and impairment. The SDQ impact supplement will be completed by the blinded assessor in an interview with the guardian and the child/adolescent (through videoconference or telephone call).

Irritability is a quite common psychopathological trait in youth and is related to many disorders, including depression and anxiety [44]. Therefore, we will evaluate the changes in child/adolescent irritability, as reported by the parents/guardians, between T0 and T1, as well as between T0 and T2. To that end, the blinded assessor will apply the parent-report Affective Reactivity Index (ARI), an index created to assess irritability [45], in an interview (by videoconference or telephone call).

Participants ≥ 12 years of age with access to a smartphone will complete an ecological momentary assessment (EMA), which is a brief in-the-moment assessment of emotions, mood, stress and anxiety. Each of those participants will receive a notification on their smartphone to answer a series of questions intended to reflect their current state (“Right now, I feel…”), with a 7-point Likert scale ranging from 0 (“not at all”) to 7 (“very much”). The EMA employs the experience sampling method (ESM) schedule [46], which consists of 17 questions and takes 3 min or less to complete. The ESM schedule will be delivered at four random times every day, within four 3-h blocks from 9:00 to 21:00, for six consecutive days in the week prior to the trial period and in the week after its end.

Participants ≥ 12 years of age with access to a smartphone will be asked to install an application designed to passively collect data from the smartphone sensors—the passive remote measurement technology (pRMT) application from the Remote Assessment of Disease and Relapse (RADAR)-base platform [47]. The pRMT application will run in the background, requiring minimal input from participants, and will use the smartphone sensors to collect data on the following: ambient noise and light; relative GPS location (i.e. the distance travelled, rather than absolute coordinates or precise geographical location); the number of Bluetooth-enabled devices nearby; the duration of calls; the number of text messages and emails; the time spent using the smartphone; the time spent on social media; and battery life. Passive data collection will take place from T0 to T2. The aim of these measures is to determine whether parameters such as physical activity (estimated by the distance travelled) and proximity to other people (estimated by analysing Bluetooth connectivity with other smartphones) might be related to changes in anxiety and depressive symptomatology and whether those parameters show any improvement after the trial.

To assess the level of parental satisfaction with the telepsychotherapy sessions (intervention group only), parents will be asked to complete an adapted version of the Telemedicine Satisfaction Questionnaire (TSQ) [48].

Potential harms were defined as the emergence of suicidal ideation and worsening of the quality of family relationships. As previously mentioned, we will use the parent-report four-item MFQ-SI to assess suicidal ideation in the last 2 weeks [31, 32], comparing T1 with T2. The MFQ-SI will be completed by the blinded assessor in an interview with the guardian (through videoconference or telephone call). To identify worsening of the quality of family relationships in the last 2 weeks, we will use the family relationship subscale of the family impact module of the Pediatric Quality of Life Inventory (PedsQL) [49]. The PedsQL will be completed by the blinded assessor in interviews with the guardian at T0, T1 and T2. The family relationship subscale of the PedsQL comprises five items, covering topics such as “lack of communication between people in my family”. For each item, there is a 5-point Likert scale (responses ranging from “never” to “almost always”).

### Participant timeline {13}

**Fig. 1:**
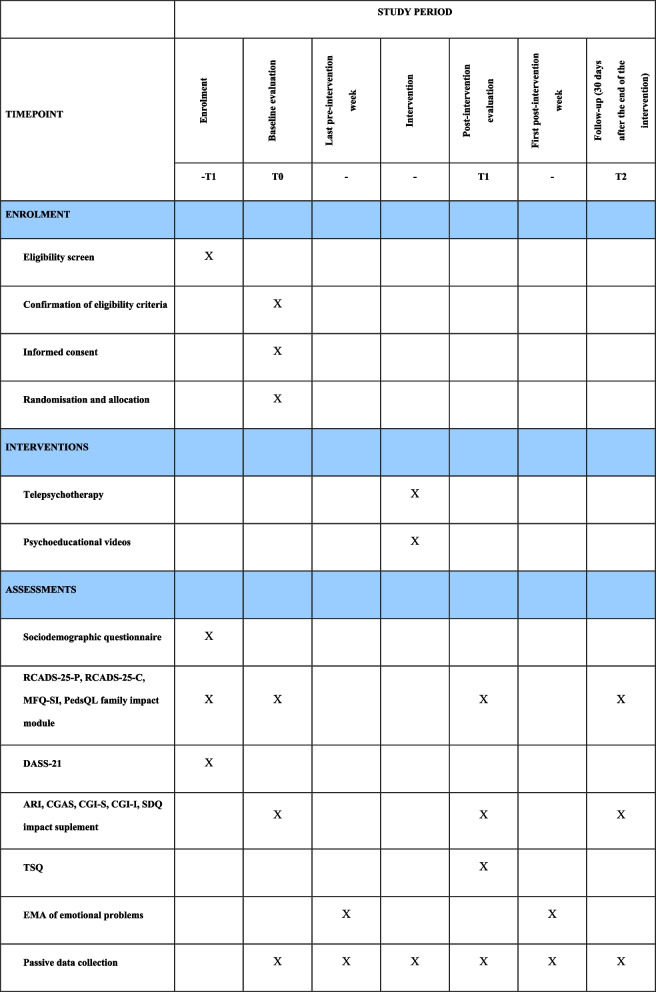
Schedule of enrolment, interventions and assessments. ARI: Affective Reactivity Index; CGAS: Children’s Global Assessment Scale; CGI-I: Clinical Global Impression-Improvement; CGI-S: Clinical Global Impression-Severity; DASS-21: 21-item Depression, Anxiety and Stress Scale; EMA: Ecological momentary assessment; MFQ-SI: Mood and Feelings Questionnaire-Suicidal Ideation; PedsQL: Pediatric Quality of Life Inventory; RCADS-25-C: 25-item version of the Revised Children’s Anxiety and Depression Scale, child report; RCADS-25-P: 25-item version of the Revised Children’s Anxiety and Depression Scale, parent report; SDQ: Strengths and Difficulties Questionnaire; TSQ: Telemedicine Satisfaction Questionnaire

All assessments (filling out electronic forms, videoconferencing and EMA) will be made via the internet or by telephone. Figure 1 shows the instruments and the time points at which they will be used.

Parents will complete an online form for eligibility screening and for the collection of sociodemographic data, as well as the 21-item parent-report psychopathology questionnaire Depression, Anxiety and Stress Scale (DASS-21) [50]. If their child/adolescent is considered eligible in this first assessment, a videoconference with a blinded outcome assessor will be scheduled. In this first videoconference, the eligibility criteria will be checked with the guardian and the child/adolescent. The baseline (T0) assessment will be made, confirming or correcting clinical data previously reported by the guardian at the eligibility screening. Two other videoconferences, in which similar clinical data will be collected by the blinded outcome assessor, will be scheduled for T1 and T2.

As previously mentioned, active smartphone data collection (the EMA) will be performed in the week prior to the intervention and in the week after its end, whereas passive smartphone data collection (through the pRMT application) will take place from T0 to T2. At T1, guardians in the intervention group will be asked to complete an online form comprising an adapted version of the TSQ [48].

### Sample size {14}

In a meta-analysis, Vigerland et al. [51] found the estimated size-effect for internet-delivered CBT in children and adolescents to be 0.62 (95% CI 0.41–0.84). Given that most of the studies assessed in that meta-analysis evaluated interventions involving a greater number of sessions than that to be evaluated in our study, we decided to be conservative and expect an effect size within the lower bound of that confidence interval, that is, 0.41. Therefore, the minimum sample size would be 190 participants, with an alpha of 0.05, power of 80% and 1:1 randomisation. Assuming an estimated attrition rate of 30%, we will need a total sample of 280 participants.

### Recruitment {15}

The study will be promoted via social media and email campaigns. People residing anywhere in Brazil will be able to participate. Parents interested in participating in the study will be referred to the study website, where they will be provided with further information regarding the study and the inclusion/exclusion criteria. They will then be asked to complete a basic online screening form and, if they meet the initial criteria, will be invited to a baseline assessment to be conducted through videoconference, in which the inclusion and exclusion criteria will also be confirmed.

## Assignment of interventions: allocation

### Sequence generation {16a}

Randomisation will occur in real time, via a computer algorithm created to minimise differences between the two groups regarding sex and age, the latter being dichotomized as 8–11 years (children) or 12–17 years (adolescents). This algorithm uses the Aitchison distance to stratify the groups according to the chosen parameters [52], as has been done in previous studies [53–55].

### Concealment mechanism {16b}

Participants meeting the inclusion and exclusion criteria will be listed sequentially by a research manager in a spreadsheet that will be used by another research manager for the randomisation. The latter research manager will have no direct contact with the patients.

### Implementation {16c}

A research manager will randomise the patients using the computer algorithm described above. Another manager will then assign the participant to the respective intervention group, in accordance with the result of the randomisation.

## Assignment of interventions: blinding

### Who will be blinded {17a}

The outcome assessor will be blinded to the group allocation. Families will be instructed not to provide the outcome assessor with any details regarding the treatment.

### Procedure for unblinding if needed {17b}

The design of the trial is single-blind. Because only the outcome assessors will be blinded, no unblinding will occur.

## Data collection and management

### Plans for assessment and collection of outcomes {18a}

All assessors will be clinical psychologists with previous experience in child and adolescent mental health who have been trained in the study protocol by the study team coordinators. The instruments used in the assessments are described below.

- Sociodemographic questionnaire—the guardian will complete an online form designed to collect data on socioeconomic status, level of education, occupation and income, as well as data such as the age/sex of the child/adolescent, COVID-19 exposure history and routines (including study hours, sleep habits, sleep quality and screen time). Sleep quality will be assessed with an adapted version of the Single-item Sleep Quality Scale, which is comparable to other sleep scales such as the Pittsburgh Sleep Quality Index [56]. Guardians will rate the sleep quality of their child/adolescent according to a 5-point Likert scale ranging from 0 (“terrible”) to 4 (“excellent”).
- DASS-21 [50]—The DASS-21 is a 21-item scale covering internalising symptoms in depression, anxiety and stress domains over the last 2 weeks. It consists of 21 statements (e.g. “I found it hard to wind down”) that will be scored by the guardians using a 4-point Likert scale ranging from 0 (“did not apply to me at all”) to 3 (“applied to me very much or most of the time”). It has been shown to possess adequate validity and reliability in the Brazilian population (Cronbach’s alpha: 0.92 for the depression subscale; 0.90 for the stress subscale; and 0.86 for the anxiety subscale) [57].
- RCADS-25—The RCADS-25 is a shortened version of the Revised Child Anxiety and Depression Scale. The original scale has 47 items and has been shown to be appropriate for use in various cultures, including that of Brazil [58]. The 25-item version is comparable to the original scale, and its use is preferred to reduce the burden of the respondent [30]. As previously mentioned, we will apply the RCADS-25-P and RCADS-25-C [28, 29]. In a clinical sample, the alpha coefficients for the RCADS-25-C depression and anxiety scales were 0.80 and 0.91, respectively, whereas they were 0.79 and 0.76, respectively, in a community sample [29]. For the RCADS-25-P, the reported alpha coefficients were 0.83 for the depression scale, 0.85 for the anxiety scale and 0.85 for the total scale [28].
- MFQ-SI [31, 32]—The original MFQ is a 33-item questionnaire on mood symptomatology that has been adapted for use in Brazil [59]. Guardians will answer only the questions on the four-item composite version related to suicidal ideation (MFQ-SI), a topic that is not covered on the RCADS-25.
- PedsQL—The PedsQL family impact module is a 36-item parent-report questionnaire that aims to assess the impact of chronic medical conditions on the quality of life of the family [49]. It has been adapted for use in Brazil [60]. We will use its five-item family relationships subscale to assess family dynamics. The subscale employs a 5-point Likert scale with responses ranging from “never” to “almost always” on topics such as “lack of communication between people in my family”.
- SDQ—The original SDQ is a 25-item questionnaire on child and adolescent psychopathology that has been adapted to many languages and used extensively in various countries, including Brazil [43], in its informant-report and self-report forms. There is an extended version of the SDQ [61] that includes a brief impact supplement (comprising five questions) designed to assess the potential burden related to the psychopathology of a child in areas such as home life and academic performance. For this study, we will use only the SDQ impact supplement, in order to assess the burden of the depressive and anxiety symptomatology.
- ARI—The ARI is a seven-item irritability scale that includes six items related to symptoms related to irritability and one item related to the potential impact of irritability [62]. The ARI has been adapted for use in Brazil [45].
- TSQ [48]—Parental satisfaction with the telepsychotherapy sessions will be assessed (in the intervention group only) with an adapted version of the TSQ [48]. It is a 14-item questionnaire that assesses parameters such as difficulties in contacting the therapist and willingness to use telemedicine services again.
- EMA of emotional problems—We will deliver an ESM protocol to assess mood, stress and emotion (Table 1). The protocol will be delivered using the COMVC application, which will send notifications for participants to rate affirmations about their current state (e.g. “Right now, I feel happy”) using an 8-point Likert scale with responses ranging from “not at all” to “very much”. It represents a brief version of the ESM protocol used in the Remote Assessment of Disease and Relapse in Major Depressive Disorder study [47], a prospective multicenter study to determine the usability, acceptability and feasibility of remote collection of data regarding the symptoms of depression.
- pRMT—The pRMT application, from the RADAR-base platform [47], will be used in order to monitor the following smartphone features: the position sensor, which allows the relative location to be registered by using data on latitude and longitude; the movement sensors (accelerometer, gyroscope and magnetometer), which allow movement to be registered by measuring acceleration, rotation and the raw magnetic field, as well as by counting steps; environmental (light) sensors, which register the level ambient light; and activity logs, which include the time spent using the smartphone in general and on specific applications, as well as battery level and nearby Bluetooth-enabled devices.

**Table 1:** Ecological momentary assessment protocol

“We would like to know how you are feeling right now”								
	**0 (not at all)**	**1**	**2**	**3**	**4**	**5**	**6**	**7 (very much)**
1. I sleep well								
2. I feel cheerful								
3. I feel down								
4. I feel anxious								
5. I feel relaxed								
6. I feel irritated								
7.I feel stressed								
8. I feel content								
9. I feel insecure								
10. I feel hopeful								
11. I feel lonely								
12. I am satisfied with myself								
13. I feel restless								
14. I feel self-confident								
15. Overall, I feel well								
16. I am ruminating								
17. I am able to concentrate well								

### Plans to promote participant retention and complete follow-up {18b}

If a participant in the intervention group misses a session, the therapist will try to reschedule it, respecting two conditions: there should be at least 48 h between sessions (so that participants can have enough time to do the “homework tasks”), and there should be no more than 4 weeks between the date of the first scheduled session and that of the last scheduled session. If the sessions cannot be rescheduled according to those conditions, the first four sessions will be prioritised, given that the fifth session is expected to be mostly a revision session. Participants will complete outcome assessments at T1 and T2, irrespective of the number of sessions completed or videos watched.

### Data management {19}

All data from the assessments and the sessions will be entered in the Research Electronic Data Capture [63], which is a secure database. The clinical data collected in the enrolment phase will be confirmed or corrected in the baseline assessment. All data, except the sociodemographic data, the TSQ score, the EMA results and the passive data, will be collected by a trained assessor.

The self-report data will be stored on the COMVC servers. The mobile smartphone sensor data will be stored on the RADAR-base platform. Both categories of data will also be backed up to Google Drive and Dropbox, and only the research team will have access to them.

### Confidentiality {27}

Information will be collected in a manner that protects the privacy of the participants. Sensitive information that might identify participants (name, hospital record number, telephone, physical address or electronic address) will not be included in the database. Each participant will be assigned an anonymous identifier. Passive data collection will also be conducted in a manner that protects sensitive data. The RADAR-base platform automatically converts information from position sensors (GPS data) into relative location using an unspecified point as a reference. Participant addresses or geographical (absolute) locations will not be registered. Likewise, we will not register the contents of emails and messages, nor the sites or content viewed on the web. Participants may choose not to participate in the EMA and the passive data collection, while still participating in the rest of the study.

### Plans for collection, laboratory evaluation and storage of biological specimens for genetic or molecular analysis in this trial/future use {33}

This trial will not involve the collection of biological specimens.

## Statistical methods

### Statistical methods for primary and secondary outcomes {20a}

We will use generalised linear mixed models (GLMMs) to analyse the effects of the intervention. The primary outcome will be the change, from T0 to T1, in the total scores on the RCADS-25-P and RCADS-25-C. Fixed and random effects will be modelled with GLMMs. The fixed effects will be the randomisation status (intervention group vs. active comparator group) as an independent variable, along with the potential confounders (age, sex and other baseline characteristics). The random effect will be on the subject (first) level. Time will be inserted as a continuous independent variable. We will use the intention-to-treat approach—all randomised subjects will be included in the analysis, even if they drop out or switch arms. The marginal means of the models will be extracted by randomisation status and time, after which they will be plotted in a time series. In a second step, we will analyse the effects that the intervention has on the symptoms of depression and anxiety in the children and adolescents. For all models, we will present the beta coefficients with a confidence interval of 95%. For all statistical tests, the level of significance will be set at 5%. Analyses will be performed with the Stata software package, version 16.0 (StataCorp, College Station, TX, USA), and the programme R, version 4.0.1 (The R Foundation for Statistical Computing, Vienna, Austria).

### Interim analyses {21b}

There will be no interim analyses.

### Methods for additional analyses (e.g. subgroup analyses) {20b}

Subgroup analyses will be conducted for children and for adolescents, as well as for the participants who were considered completers (i.e. attending three or more sessions) and did not start other mental health treatments (e.g. another psychotherapy intervention or psychotropic medication) during the intervention period.

### Methods in analysis to handle protocol non-adherence and any statistical methods to handle missing data {20c}

For the intention-to-treat analysis, we will use GLMMs. We will also perform secondary analyses excluding those not considered completers and those who started any other mental health treatment during the intervention period.

### Plans to give access to the full protocol, participant-level data and statistical code {31c}

Any data required to support the protocol can be supplied on request. The datasets analysed during the current study are available from the corresponding author on reasonable request.

## Oversight and monitoring

### Composition of the coordinating centre and trial steering committee {5d}

The trial coordinating centre comprises the principal investigator and trial managers. They will monitor the progress of the trial (including the recruitment of participants and attrition) and ensure adherence to its protocol. They will report any adverse events to the trial steering committee and research ethics committee.

The senior investigator of our group chairs the trial steering committee. He will therefore be responsible for decisions regarding the continuation, modification or termination of the study. Modifications will be implemented only after approval by the research ethics committee and adjustment of the clinical trial registry.

### Composition of the data monitoring committee, its role and reporting structure {21a}

This protocol has been approved by the local research ethics committee, which did not recommend the creation of a data monitoring committee.

### Adverse event reporting and harms {22}

The risk for participants is expected to be minimal, because the intervention is non-invasive. However, we will monitor participants to identify any potential side effects of the intervention, including increased symptomatology and emergence of suicidal ideation. In the clinical assessments (interviews with the guardians and the children/adolescents), the blinded assessor will collect data on symptomatology (through the use of the RCADS-25-P, ARI, SDQ impact supplement, CGI-S and CGI-I), emergence of suicidal ideation (through the use of the MFQ-SI) and disruption of family dynamics (through the use of the PedsQL family impact module). The blinded assessor will also collect data on any other form of mental health treatment that the family seeks between T0 and T2, including psychotherapy with other providers or psychopharmacological treatment.

If by the end of treatment the assessor identifies a significant level of symptomatology, the child/adolescent will be referred to continue the treatment within the Brazilian public health care system. One of the child psychiatrists on the team will evaluate the child/adolescent if necessary.

### Frequency and plans for auditing trial conduct {23}

The trial coordinating team will meet weekly to monitor the progress of the trial. The trial steering committee will oversee the trial on a monthly basis.

### Plans for communicating important protocol amendments to relevant parties (e.g. trial participants, ethical committees) {25}

Meetings of the research ethics committee meetings might be scheduled if any adverse event occurs, or in the event that the trial steering committee decides to make amendments to the trial protocol. Amendments to the protocol will be made only after approval by the research ethics committee. Should any amendment be made, the clinical trial registry (https://clinicaltrials.gov/ct2/show/NCT05139433) will be updated.

### Dissemination plans {31a}

At the end of the study, we intend to publish the data in a peer-reviewed journal and make the intervention manual and the psychoeducational videos public.

## Discussion

Videoconferencing in mental health was first employed approximately 70 years ago, in the 1950s [27], and its use increased considerably during the COVID-19 pandemic [64]. However, despite the growing body of literature on the topic, there is still a scarcity of scientifically validated protocols focusing on telepsychotherapy for children and adolescents [27]. If proven effective, the present protocol could not only help increase the evidence in this area but also represent a tool to improve access to mental health care even outside the context of the pandemic. The intervention is described in detail in the manual, which should facilitate its reproduction in other contexts. It is based on cognitive-behavioural techniques, some of which have previously been tested in crisis situations [65]. We have adapted some of those strategies to take advantage of new technologies, such as the delivery of psychoeducational videos via the internet.

One aspect of our study that is secondary but still of great relevance is the use of the smartphone as a tool to collect participant data, which is expected to increase ecological validity and reduce memory bias [66]. The use of passive data collection by the pRMT application might allow us to draw interesting correlations between mental health symptomatology and aspects such as screen time and physical activity.

Our protocol has some limitations. For instance, the active control group intervention is a self-guided psychoeducational activity, which could result in an overestimation of the intervention group results. We will not be comparing the intervention to a therapist-lead intervention to test the specific effects of the implementation procedure. The reportedly high (approximately 30%) dropout rates in trials of psychotherapy interventions [67–70] might be a challenge. The number of sessions (five) might also be insufficient for individuals who are more symptomatic. It is estimated that three quarters of the Brazilian population has internet access, although the level of connectivity is not homogeneous, with some areas lacking stable connections [71]. These factors could therefore limit the access to the intervention to part of the population. However, it is a robust intervention that could help increase the existing evidence in the area of telemedicine and in alternative ways of measuring data, such as via smartphone apps. It also foresees the alternative use of telephone for the intervention, in cases where poor internet connectivity continues to be an issue.

## Trial status

Version 1.0 of the protocol was registered on November 17, 2021. Minor amendments (reducing the number of primary outcomes) were made and registered in July 2022. Recruitment began in October 2021 and is expected to end in January 2023.

## Data Availability

Any data required to support the protocol can be supplied on request. The datasets analysed during the current study are available from the corresponding author on reasonable request.

## Abbreviations

ARI: Affective Reactivity Index
CBT: Cognitive-behavioural therapy
CGAS: Children’s Global Assessment Scale
CGI-I: Clinical Global Impression-Improvement
CGI-S: Clinical Global Impression-Severity
DASS-21: 21-Item Depression, Anxiety and Stress Scale
EMA: Ecological momentary assessment
ESM: Experience sampling method
GLMM: Generalised linear mixed model
MFQ: Mood and Feelings Questionnaire
MFQ-SI: Mood and Feelings Questionnaire-Suicidal Ideation
PedsQL: Pediatric Quality of Life Inventory
pRMT: Passive remote measurement technology
RCADS-25-C: 25-Item version of the Revised Children’s Anxiety and Depression Scale, child report
RCADS-25-P: 25-Item version of the Revised Children’s Anxiety and Depression Scale, parent report
SDQ: Strengths and Difficulties Questionnaire
TSQ: Telemedicine Satisfaction Questionnaire
